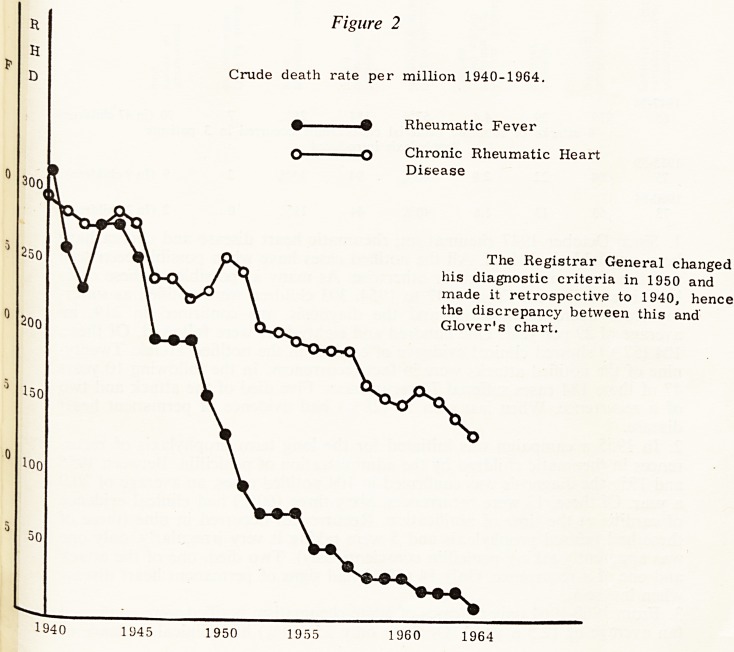# On the Changing Face of Rheumatism

**Published:** 1967-04

**Authors:** C. B. Perry


### ON THE CHANGING FACE OF RHEUMATISM

BY

PROF. C. B. PERRY

Ever since the turn of the century the incidence and severity of acute rheumatism have been steadily falling. In 1889 Cheadle stated that acute rheumatism was the commonest cause for admission to hospital and was also frequently seen in private practice. A similar state of affairs is revealed by the figures for admission to one ward at the Bristol Children's Hospital in the charge of Dr. Edgeworth who was not particularly interested in acute rheumatism. From June 1890 to July 1893 there were 197 admissions and of these 57 (29%) were for rheumatism or chorea. By 1925 Hutchison could say " that it was a disease of hospital and not consulting practice and that he could count on one hand the cases of acute rheumatic carditis that he had seen in consul-

*Figure 1*

tation in the course of twenty-five years ". In the survey carried out in Bristol and the surrounding counties in 1927-1930 the incidence of rheumatic heart disease was found to be 7.72 per 1000 school children in Bristol and varied from 1.03 to 2.27 per 1000 in the surrounding counties. In 1930 Glover described it as being thirty times as common among the poor as among the well-to-do. He drew attention to the decreasing incidence and severity and in 1943 pointed out the great acceleration in this decrease that took place in the war years (Fig. 1). This fall in the death rate has continued since the war at an increasing rate. The death rate per million which was 31 in 1940 had fallen to 1 by 1964. The mortality from chronic rheumatic heart disease is falling less rapidly (Fig. 2). It is interesting to observe that the graph of the crude death rate per million for chronic rheumatic heart disease from 1940 to 1964 almost parallels that for acute rheumatism during the years 1915 to 1939 (i.e. 25 years later).

In 1937 Perry and Roberts showed that in Bristol the incidence of rheumatic heart disease was closely related to over-crowding and suggested

*Figure 2*

that this over-crowding might be an index of poverty. Further studies by Daniel (1943) and Perry (1944) confirmed the very close correlation between acute rheumatism and poverty in Bristol.

In the last two decades, the prospects for a child with acute rheumatism have been changed in the country as a whole, coincidentally with increasing standards of living, the use of antibiotics, and the general decline in severity of all streptococcal infections. In Bristol the possibility of studying the effect of these changes has been improved by the introduction of notification of rheumatism to the Health Authorities. For the purpose of studying changes in acute rheumatism in Bristol, three distinct periods have been chosen, and these are analysed below, and summarised in Table I.

TABLE I

Incidence and severity of acute rheumatism, rheumatic heart disease and chorea in Bristol 1947-1964.

| Approximate average school population 1000 | Notified cases confirmed | Average per year | Average annual incidence per 10,000 school children | Carditis clinically in notified attacks | Number successfully traced | Persistent cardiac lesion | Died of attack or recurrence | Recurrences known to date |
|---|---|---|---|---|---|---|---|---|
| 1947-54 | | | | | | | | |
| 65 | 219 | 29 | 4.4 | 57% | 184 | 28% | 7 | 70 (in 47 children) |
| | 4 attacks of subacute bacterial endocarditis occurred in 3 patients | | | | | | | |
| | Prophylaxis introduced | | | | | | | |
| 1955-59 | | | | | | | | |
| 75 | 104 | 22 | 2.8 | 60% | 94 | 13% | 2 | 9 (In 9 children) |
| 1960-64 | | | | | | | | |
| 73 | 63 | 13 | 1.6 | 40% | 44 | 11% | 0 | 2 (In 2 children) |

1. Since October 1947 rheumatism, rheumatic heart disease and chorea have been notifiable in Bristol. All the notified cases have when possible been seen and the diagnosis confirmed or otherwise. As many as possible of these cases have been followed. From 1947 to 1954, 303 children were notified as suffering from acute rheumatism and the diagnosis was confirmed in 219, an average of 29 per year. One hundred and eighty-four were followed. Of these, 104 (57%) showed clinical evidence of carditis in the notified attack. Twenty-nine of the notified attacks were in fact recurrences. In the following 10 years 47 of these 184 cases suffered 70 recurrences. Five died of the attack and two of a recurrence. When last seen 52 (28%) had evidence of permanent heart disease.

2. In 1955 a campaign was initiated for the long term prophylaxis of recurrences in rheumatic children by the administration of penicillin. Between 1955 and 1959 the diagnosis was confirmed in 104 notified cases, an average of 20.9 a year. Of these, 13 were recurrences. Sixty-three (60%) had clinical evidence of carditis at the time of notification. Recurrences occurred in nine (three of these had refused prophylaxis and 5 were taking it very irregularly, only one was apparently taking penicillin conscientiously). Two died, one of the attack and one of a recurrence. Only 14 (13%) had signs of permanent heart disease when last seen.

3. From 1960-1964 only 63 cases of acute rheumatism notified were confirmed (an average of 12.5 a year). Of these only 25 (40%) had clinical evidence of carditis. None died. It was possible to follow up forty-four. Among these there were two recurrences (one in a child apparently receiving prophylaxis regularly and one in a defaulter). When last seen only 5 (11%) had signs of permanent heart disease.

A similar fall in incidence and severity has been recorded in U.S.A. The death rate from rheumatic fever which was 10.2 per 100,000 in 1920 had fallen to 4 per 100,000 in 1960.

### REFERENCES

Cheadle, W. B., (1889) *Lancet,* **i**, 821, 871 and 921.
Daniel, G. H. (1942), *Journ. Roy Stat. Soc.*, 105, 197.
Glover, J. A. (1930), *Lancet* **i**, 499, (1943) *Lancet* **ii**, 51.
Perry, C. B. (1944) *Brist. Med-Chi. Journal.* **61**, 1.
Perry, C. B. & Roberts, J. A. F. (1937) *B.M.J.* (supp.) **2**, 154.

## Figures and Tables

**Figure 1 f1:**
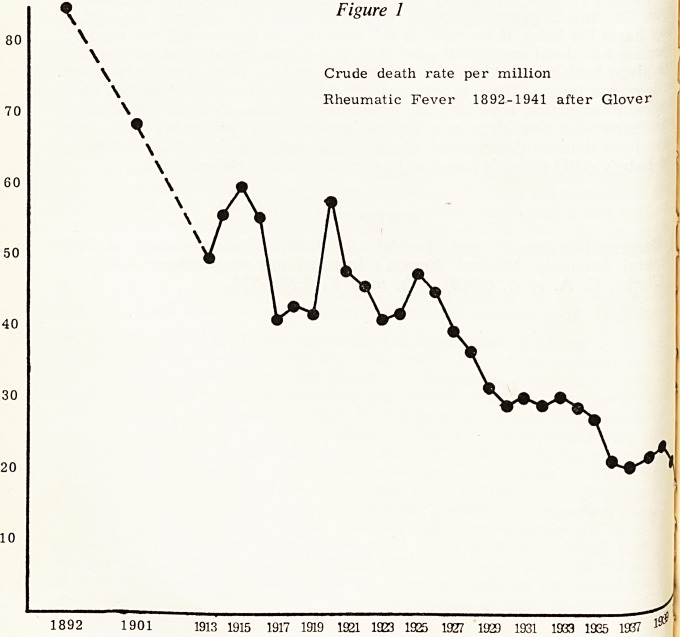


**Figure 2 f2:**